# ATYPICAL DEBUT OF BIPOLAR DISORDER IN AN ADOLESCENT: POST-COVID SYNDROME, PARANEOPLASTIC SYNDROME, OR SOMETHING ELSE?

**DOI:** 10.1192/j.eurpsy.2023.1620

**Published:** 2023-07-19

**Authors:** I. Ezquiaga Bravo, R. M. Cámara, I. Gómez, A. Vilar, A. Rodríguez, M. T. Nascimento, S. Batlle, L. M. Martín

**Affiliations:** INAD, Parc de Salut Mar, BARCELONA, Spain

## Abstract

**Introduction:**

Paraneoplastic syndromes (PNS) can be expressed with a wide variety of neurological and psychiatric symptoms: alterations in consciousness, cognition, behaviour, mood or perception. Testicular tumours have been related to different expressions of PNS, but, to date, no relationship with bipolar disorder has been described.

On the other hand, the relationship between SARS-CoV2 infection and subsequent affective conditions has also been recently described. Between 30-40% of people affected by the infection present symptoms of depression in the following months.

**Objectives:**

To describe a case of a 17-year-old patient with an atypical onset of bipolar disorder a few months after a SARS-CoV2 infection and a few months before a testicular germ cell tumour was detected.

**Methods:**

Description of a clinical case, its differential diagnosis and the literature review associated.

**Results:**

This is a 17-year-old adolescent with no previous psychiatric history, who is referred to a day centre after committing a suicide attempt. The patient presented an average premorbid functioning. Stands out, a SARS-CoV2 infection 3 months before the onset of symptoms. He presents repeated and self-limited episodes (maximum 3 weeks) of major depressive symptoms: autolytic ideation, hypothymia, asthenia, clinophilia, isolation, anhedonia, mutism, psychomotor retardation, lack of hygiene, hyporexia, hypersomnia; that alternates with periods of stability and with others of symptoms of hypomania (sudden improvement in mood, increased activity and plans), also lasting a few days. Paradoxic response to treatment with antidepressants, presenting irritability and exacerbation of suicide ideas. Good tolerance and response to treatment with low doses of aripiprazole and quetiapine. The patient was diagnosed as type II bipolar disorder with rapid cycling.

A few days after definitive diagnosis, a testicular germ cell tumour was detected, for which he had to undergo surgical intervention and chemotherapy treatment. At this point, it is suggested that the symptoms could be included in a paraneoplastic condition prior to the tumour. Months after the remission of the cancer, the patient does not present symptoms of relapse or metastasis, but mood swings persist, of lesser intensity, every few weeks. Treatment with lamotrigine was started at increasing doses, with good response and tolerance from the start.

**Conclusions:**

The onset of mental health disorders in adolescents can be complicated by the non-specific or atypical early or prodromal symptoms. This degree of complexity increases when somatic pathologies coexist and even more if those pathologies have yet to be fully understood and studied, such as paraneoplastic syndromes or SARS-CoV2 infections. It is necessary to continue investigating the interrelationship between somatic and psychiatric conditions in order to provide more specific and rapid clinical responses.

**Disclosure of Interest:**

None Declared

